# Trends of cholera epidemics and associated mortality factors in Cameroon: 2018–2023: a cross-sectional study

**DOI:** 10.1186/s12889-025-23007-5

**Published:** 2025-05-16

**Authors:** Akenji Blaise Mboringong, Sen Claudine Henriette Ngomtcho, Roland Ndip Ndip, Esso Endalle Linda, Dibog Luc Bertand, Mendjime Patricia, Belinga Sandrine, Balana Flore Essiene, Theodore Ntamack, Etoundi Georges Alain Mballa

**Affiliations:** 1 National Public Health Laboratory (Ministry of Public Health), Yaounde, Cameroon; 2 Department of Microbiology and Parasitology, Faculty of Science, University of Buea, Buea, Cameroon; 3 Department for the Control of Diseases, Epidemics and Pandemics (Ministry of Public Health), Yaounde, Cameroon; 4 University of Dschang, Dschang, Cameroon; 5 Department of Biotechnology and Food Technology, Faculty of Science, University of Johannesburg, Johannesburg, South Africa

**Keywords:** Cholera, Epidemiology, Associated factors, Mortality

## Abstract

**Background:**

Cameroon has faced frequent and severe cholera outbreaks since 1971, with case-fatality rates (CFRs) ranging from 12% in 1991, to 5.3% in 2014, all higher than the less than 1% cholera CFR target set by WHO. However, not many studies providing insight on context-specific risk factors have been published. The purpose of this study was to describe the recent cholera outbreaks in Cameroon and to determine factors associated with mortality.

**Methods:**

This was an analytical cross-sectional study that employed a retrospective design exploiting Ministry of Public Health cholera line-lists from 2018–2023. These line lists were obtained from the Public Health Emergency Operations Coordination and Control Center, compiled into a single Microsoft Excel Sheet, cleaned and analyzed using Microsoft Excel 2016 and SPSS version 20. Cholera cases were defined as those confirmed in reference laboratories via stool culture and suspected cases with proven epidemiological link to laboratory-confirmed cases (suspected cases in health districts with active laboratory-confirmed cases). Factors associated with cholera mortality were identified using binary logistic regression (adjusted odds ratios), after socio-demographic, clinical, and geographical distribution of cholera cases were described. Maps were generated using QGIS version 3.28.14.

**Results:**

Between May 2018 and March 2023, Cameroon experienced four cholera epidemics resulting in 18,986 reported cases and affecting 8 out of 10 administrative regions. The three coastal regions (Littoral, South and South-West Region) reported 83.4% (15,839/18,986) of all the cases while the remaining five affected regions jointly reported 16.6% (3,147/18,986) cases. The most represented age group were those aged 25–35 years (21.9%; 4,163/1,876) and the male: female sex ratio was 1.27. The overall CFR was 2.7% (478 deaths/17,967 cases with known outcome) and was highest among persons > 65 years (6.8%; 59/869). Urban areas notified more cases than rural areas (13,267 vs 5,484). Factors associated with increased mortality were male sex (aOR 1.61, *95% CI: 1.30—2.04*), dry season (aOR 1.67, *95% CI: 1.28—2.22*), age above 45 years (aOR 1.79, *95% CI: 1.45—2.22*) and severe dehydration at consultation (aOR 12.76, *95% CI: 7.66–21.25*).

**Conclusions:**

Cholera outbreaks occurred in eight out of the ten administrative regions in Cameroon during the study period and mortality appeared to be driven by multiple factors notably severe dehydration at time of consultation, advanced age, male sex and the dry season. The high caseloads and case-fatality rates reiterate the need for further strengthening of existing cholera surveillance and outbreak response mechanisms.

**Supplementary Information:**

The online version contains supplementary material available at 10.1186/s12889-025-23007-5.

## Introduction

Cholera is an acute diarrheal infection caused by ingestion of food or water contaminated with the bacterium *Vibrio cholerae*. There are many serogroups of *V. cholerae*, but only two – O1 and O139, cause epidemics in the human population [1]. *Vibrio cholerae* can survive in fresh-water such as lakes and salt-water (coastal, estuarine areas) [1–3]. Cholera tends to follow seasonal patterns, often exacerbated by environmental factors such as high levels of precipitation [4–7]. While some studies have clearly demonstrated a link between environmental exposure and illness in humans, once introduced into the human population, interhuman transmission may predominate [8–11].

Despite having been cholera-free before 1970, Africa now bears the brunt of the cholera burden [12]. Cameroon reported its index cholera cases in February 1971 [13]. This was followed by a 20-year period characterized by sporadic disease clusters. The cholera burden in Cameroon has increased during the past three decades, with 4026 cases notified in 1991, 5796 in 1996, 8005 in 2004, 10,759 in 2010 and 3355 in 2014 [14, 15]. Previously identified risk factors for cholera infection in Cameroon include poor hygiene, age below 21 years, eating outside the home and poor food storage practices [16]. Meanwhile, increased mortality has been linked with delays in case-finding and with difficulty of access to standard care including non-use of a cholera treatment center, receiving health care in a temporary community-treatment center instead of a hospital, and greater than 4 h delay between the onset of symptoms and the decision seek treatment in a treatment center [17]. However, despite these challenges, the case-fatality rate (CFR) during cholera outbreaks decreased progressively from 12% in 1991, to 5.3% in 2014, most probably due to improved detection and case-management strategies [15, 18].

Previous studies have examined risk factors during earlier outbreaks in Cameroon [19], but not for the 2018–2023 outbreaks. Consequently, there is a paucity of information about the geographical distribution of recent outbreaks as well as context-specific risk factors of the disease in Cameroon. Filling these knowledge gaps is essential for the formulation and implementation of well-adapted cholera control strategies and was the purpose of this study.

## Methods

### Study setting

Cameroon is located in Central Africa along the Atlantic coast. It has ten administrative regions, with the southern regions located within the equatorial rain-forest, the northern regions in the southern part of the Sahara Desert, and the regions in-between are characterized by savanna. Three of the regions within the equatorial rainforest (Littoral Region, South Region and South-West Region) have long coastlines along the Atlantic Ocean. Cameroon has four climatic subzones [20]. The zone spanning most of the Far-North Region up to the Lake Chad Basin comprises the Sudano-Sahelian zone, the North and Adamawa Regions comprise the Tropical humid zone, most of the North-West, South-West and West Region fall within the Equatorial Monsoon zone, while the Center, South and East Regions comprise the Guinea Equatorial zone [20]. Each zone has varying lengths and numbers of rainy and dry seasons with the Guinea Equatorial subzone experiencing the highest average annual rainfall (1500 to 2000 mm) while the Sudano-Sahelian subzone has the shortest rainy season with an average annual rainfall (400 to 900 mm) [19]. The ten administrative are divided into 200 health districts and further into 1962 health areas [21]. Health districts are operational administrative units that are tasked with implementing the strategies of the ministry of health (vaccination, response to disease outbreaks, general patient care, child deliveries). Each health district has at least one public hospital (known as a district hospital), several private-sector clinics and hospitals, and serves 50,000 to 75,000 people (rural districts) or 100,000 to 150,000 people (urban districts). Health areas (located within the health districts) each have at least a public health center which could be a medicalized health center or an integrated health center, meant to further provide essential decentralized health services such as vaccinations, child deliveries and management of some diseases [22, 23]. Recent data suggests that the urban population in Cameroon is higher than the rural population: 58.2% versus 41.8% [24]. Figure 1 (additional File 1) shows the administrative regions and the health districts in Cameroon. It also indicates administrative regions and health districts that were affected by cholera during the study period.

In Cameroon, access to safe water is approximately 70% nationally, but with a gap between urban (82%) and rural (52%) coverage [25]. Basic sanitation access is even lower, at 43% overall, with a stark difference between urban (58%) and rural (22%) access [25]. These challenges are worsened by humanitarian crises: the CAR conflict, the Northwest/Southwest crisis, and the Boko Haram insurgency in the Far-North have increased internal displacement, further reducing access to safe water in various regions and districts [25].

Three large humanitarian crises are on-going in Cameroon (the Boko-Haram insurgency in the northern regions, the North-West and South-West armed conflict, and the Central African Republic refugee crisis that has led to an influx of refugees into Cameroon’s East, Adamawa and North Regions) [26]. By February 2023, the total number of internally-displaced persons were approximately 427,833 persons in the Far-North Region, over 375,000 in the North-West and South-West Regions and over 329,000 refugees from the Central African Republic into the above-mentioned regions [26]. Though official counts are not available, it is reported that cities like Douala, Limbe, Buea, and Yaoundé harbor the majority of the internally-displaced persons from the North-West and South-West armed conflict.

### Study design

This was an analytical cross-sectional study that employed a retrospective design and utilized data from the Public Health Emergency Operations Coordination Centre (PHEOCC) in Yaoundé. The study was carried out from November 2022 to May 2023, exploiting cholera data from May 2018 to March 2023.

### Study population

The study population consisted of all laboratory-confirmed and suspected cases with epidemiological link to laboratory-confirmed cases of cholera recorded in the line-lists obtained from the Public Health Emergency Operations Coordination Center (PHEOCC) of the Ministry of Public Health (MOH) from May 2018 to March 2023. Laboratory-confirmed cases were all cases in the 2018 to 2023 cholera line-lists with documented stool culture positive for *Vibrio cholerae* serogroup O1 (confirmed in a national cholera reference lab) while epidemiologically-linked cases were suspected cases with no laboratory confirmation that were reported in health districts with active laboratory-confirmed cases. In resource-limited settings such as Cameroon, during large cholera outbreaks, only a fraction of cases is laboratory-confirmed due to insufficient resources. Suspected cases occurring in heath districts with laboratory-confirmed cases are considered and managed in the same way as laboratory-confirmed cases.

### Database analysis

The line-lists for the period from May 2018 to March 2023 were compiled into an Excel database, cleaned checked for completeness, duplicity and validity of variables. Socio-demographic, clinical and epidemiological data were then extracted for analysis. Epidemic curves were drawn using data on the date of onset of disease. Attack and case-fatality rates were calculated for each epidemic, administrative region and health district using the following formulae:

$$\mathbf{Cholera}\boldsymbol\;\mathbf{Attack}\boldsymbol\;\mathbf{Rate}\;=\;(\mathrm{Number}\;\mathrm{of}\;\mathrm{cholera}\;\mathrm{cases}\;\mathrm{reported}\;\mathrm{during}\;\mathrm{the}\;\mathrm{epidemic}\;\mathrm{or}\;\mathrm{within}\;\mathrm{the}\;\mathrm{health}\;\mathrm{district}/\mathrm{administrative}\;\mathrm{region})\;\div\;(\mathrm{Total}\;\mathrm{population}\;\mathrm{at}\;\mathrm{risk}\;\mathrm{in}\;\mathrm{the}\;\mathrm{involved}\;\mathrm{health}\;\mathrm{district}/\mathrm{administrative}\;\mathrm{region})\;\times\;100,000$$

$$\mathbf{Cholera}\boldsymbol\;\mathbf{Case}\boldsymbol-\mathbf{Fatality}\boldsymbol\;\mathbf{Rate}\boldsymbol\;\boldsymbol(\mathbf{CFR}\boldsymbol)\;=\;(\mathrm{Number}\;\mathrm{of}\;\mathrm{cholera}\;\mathrm{deaths}\;\mathrm{reported}\;\mathrm{during}\;\mathrm{the}\;\mathrm{epidemic})\;\div\;(\mathrm{Total}\;\mathrm{number}\;\mathrm{of}\;\mathrm{cholera}\;\mathrm{cases}\;\mathrm{with}\;\mathrm{known}\;\mathrm{outcome}\;(\mathrm{dead}/\mathrm{alive})\;\mathrm{reported}\;\mathrm{during}\;\mathrm{the}\;\mathrm{epidemic})\;\times\;100,000$$

Case-fatality rates were also computed for age groups, sex, season (rainy or dry season) and notification zone (rural or urban area). Statistical associations between these variables and outcome (alive or dead) were evaluated.

The association between level of dehydration at time of consultation and outcome (alive or dead) was also calculated. The PHEOCC used the WHO dehydration scale to classify dehydration among cases. Mild dehydration was defined as thirst accompanied by < 5% body weight loss with no hemodynamic changes [27]. Moderate dehydration was defined as visible signs and symptoms of dehydration with mild hemodynamic changes and/or 5–10% loss of body weight [27]. Severe dehydration was defined as severe hemodynamic changes, altered consciousness and/or > 10% loss of body weight [27].

### Data sources

Seasonal data (rainy season and dry season variations per climatic subzone in Cameroon) were obtained from publications by Moise et al*.* and Cornelius et al*. *[19, 20]. Data on the estimated population sizes of health districts was obtained from the MOH and used in the calculation of attack rates [28].

### Statistical analyses

To describe geographical distribution of cases QGIS version 3.28.14 was used. Microsoft Excel 2016 for Windows was used to generate frequency tables, bar charts and epidemiological curves. Categorical variables were described using proportions while continuous data was summarized using measures of central tendency. Statistical Package for Social Science version 20 (SPSS v20) was used for inferential analyses. By means of this software, Kruskal–Wallis test was used to compare medians of continuous variables with asymmetrical distribution, followed by calculation of odds ratios and 95% confidence intervals. All variables associated with cholera mortality in bivariate analysis with a significance level of *p* < 0.20 were included in binary logistic multivariate analyses. The final multivariate model considered as significant, only variables with *p* < 0.05 for adjusted odds ratios.

During bivariate analyses, the pairwise deletion approach was used in order to minimize the introduction of bias due to absent data. The authors considered pairwise deletion simpler to implement than imputation. For multivariate analysis, only cases with complete data for all the variables were included in the model in order to improve the predictive accuracy of the model.

### Ethical and administrative considerations

This study was conducted in compliance with the Declaration of Helsinki’s guidance on the use of secondary data from human subjects which requires ethical clearance to be obtained from an ethical review board when confronted with inability to obtain consent directly from subjects whose data are obtained from stored databases. Ethical clearance was obtained from the University of Buea Faculty of Health Sciences Institutional Review Board (Reference number: 2023/1974–02/UB/SG/IRB/FHS). Authorization to use Ministry of Public Health (MOH) data was obtained from the PHEOCC in Yaounde, Cameroon. This analysis was done on an anonymized, aggregated, de-identified secondary dataset so it was not possible to involve the patients or the public in the designing, execution, or dissemination of the results of the study. Consequently, the study did not include direct participation from patients or the public.

## Results

### Distribution of cases by administrative region and health district in Cameroon (2018–2023)

Data on cholera epidemics in Cameroon from May 2018 to March 2023 was collected for this study. Between 2018 and 2023, Cameroon experienced four cholera outbreaks. The first epidemic affected only the North, Center and Far-North Regions with a total of 1,628 cases from 2018 to 2019. This epidemic affected 34 health districts (HD): eight in the Far North, thirteen in the North Region and thirteen in the Center region**.** The epicenters of this epidemic were Garoua I with 309 cases (19.0% of total notified cases), Pitoa with 213 cases (13.1% of total notified cases) and Bibemi with 209 cases (12.8% of total notified cases). These health districts are all located in the North Region.

The second epidemic was confined mainly to the Bakassi health district (359 cases) and the Ekondo-Titi health district (1 case) in the South-West Region. It occurred between November and December 2019.

The third outbreak began in the Littoral Region in January 2020 and spread to the Center, South and South-West Regions, resulting in 1,889 cases by July 2020 when it ended. It affected 18 health districts: ten in the Littoral, two in the South-West Region, three in the Center region and three in the South Region. The most affected health districts were Kribi in the South Region with 762 of the 1,889 cases (40.3%), followed by Nylon with 299/1,889 cases (15.8%) and Bonassama with 214/1,889 (11.3%) both in the Littoral Region.

The fourth epidemic began in October 2021, in the South-West Region and by March 2023, eight regions had reported cases (15,111 cases reported by March 2023), with the majority of them occurring in the Littoral Region (7,412/15,111 cases; 49.1%) and South-West Region (6,027/15,111 cases; 40.0%). This prolonged outbreak affected 61 health districts: 18 in the Center Region, 18 in the Littoral, 12 South-West Region, six in the Far North, four in the South Region, two in the East Region and one in the North Region. The distribution of cases by administrative region and health district is illustrated in Fig. 2 (Additional File 2).

The most affected districts in the Littoral Region were Bangue (485/7,412 notified cases; 6.5%), Bonassama (645/7,412 notified cases; 8.7%), Cité des Palmiers (707/7,412 notified cases; 9.5%), Deido (1,997/7,412 notified cases; 26.9%). In the South-West Region, Bakassi (309/6,027 notified cases; 5.1%), Ekondo-Titi (353/6,027 notified cases; 5.9%), Buea (935/6,027 notified cases; 15.5%) and Limbe (3,136/6,027 notified cases; 52.0%) notified cases) were the most affected. Djoungolo (337/707 notified cases; 47.7%) reported the most cases in the Center Region.

The coastal Regions (Littoral, South-West, South) accounted for 83.4% (15,839/18,986 notified cases) reported during the study period (Table 1). A total of 81/200 (40.5%) of health districts in Cameroon were affected by cholera between May 2018 and March 2023.

**Table 1:** Number of cholera epidemics, duration, and case notifications in Cameroon from May 2018 to March 2023

Period	Region	Date of onset	Date of last notification	Duration in months	No. of cases per region	Total no. of cases per epidemic
**2018–2019**	North	18/05/2018	17/12/2019	19 months	1,212	**1,628**
	Center	11/07/2018	12/08/2018		66	
	Far North	01/07/2019	23/11/2019		350	
**2019**	South-West	13/11/2019	23/12/2019	1 month	359	**359**
**2020**	Center	13/07/2020	05/08/2020	11 months	62	**1,889**
	Littoral	04/01/2020	18/12/2020		967	
	South	01/05/2020	18/10/2020		780	
	South-West	22/02/2020	19/09/2020		80	
**2021–2023**	South-West	15/10/2021	1/9/2022	17 months at the time of data collection	6,027	**15,111**
	Littoral	18/11/2021	29/12/2022		7,412	
	Center	23/10/2021	On-going		707	
	Far North	01/01/2022	15/12/2022		486	
	North	14/02/2022	16/6/2022		50	
	South	10/12/2021	15/5/2022		214	
	West	14/04/2022	3/12/2022		203	
	East	13/07/2022	On-going		12	
**TOTAL**					**18,986**	**18,986**

### Age and sex distribution of cholera cases during the 2018–2023 outbreaks in Cameroon

The most represented age group was the 25 to 35-year age group (22.0%; 4,136/18,762 cases), followed by the 15 to 25-year age group (21.4%; 4,012/18,762 cases) and the 35 to 45-year age group (14.7%; 2758/18,762 cases). The 0 to 5-year age represented 9.5% (1782/18,762) of the total caseload. The overall male: female sex ratio was 1.27 (10,594 males/8,334 females).

### Attack rates and case-fatality rates of cholera in Cameroon during the 2018–2023 outbreaks

The overall CFR was 2.7% (476 deaths/17,489 cases with known outcome). With each passing epidemic, there was a progressive decrease in the CFR: 5.0% (80 deaths/1,613 cases with known outcome) during the 2018–2019 epidemic, 4.4% during the 2019 epidemic (16 deaths/360 cases with known outcome), 4.2% (78 deaths/1,824 cases with known outcome) during the 2020 outbreak, and 2.1% (302 deaths/14,716 cases with known outcome) during the 2021–2023 epidemic. The coastal regions, which are the Littoral, South-West and South regions, recorded the highest attack rates: 412/100,000 in the South-West Region, 206/100,000 in the South Region, and 111/100,000 in the Littoral Region compared to the rest of the regions (Table 2) but conversely, had the lowest CFRs (Littoral: 2.7% (209 deaths/7,714 cases with known outcome), South: 2.8% (27 deaths/950 with known outcome); South-West: 1.7% (412 deaths/6,467 cases with known outcome). The CFR was highest among cases over 65 years old (6.8%; 59 deaths/869 cases with known outcome) and lowest for cases aged 15–25 years (1.3%; 48 deaths/3,834 cases with known outcome).

**Table 2:** Attack rates and case-fatality rates of cholera epidemics per region from 2018 to 2023 in Cameroon

Region	Total cases notified	Total population at risk	Attack rate/100000	Total Alive	Total Dead	CFR (%)
**Coastal Regions**						
South-West	6,465	1,737,783	412	6,355	110	1.7
South	994	480,552	206	923	27	2.8
Littoral	8,379	7,502,297	111	7,505	209	2.7
**Other affected Regions**						
Far North	836	1,146,878	72.9	653	35	5.1
North	1,262	267,803	47	1,182	64	5.1
West	203	775,848	27	164	7	4.1
East	12	44,683	26.9	10	2	16.7
Center	835	9,037,324	9.2	695	22	3.1
** Total/average**	18,986	23,396,168	81.2	17,489	476	2.7

### Severity of dehydration at time of consultation during the 2018–2023 cholera outbreaks in Cameroon

Twenty percent (3,311/16,550) of cases reported mild dehydration, 49% (811/16,550) had moderate dehydration and 31% (5,123/16,550) had severe dehydration at time of consultation. The proportion of cases with severe dehydration was highest among those older than 65 years (39.1%; 299/764) and lowest among the cases aged 0–5 years (21%; 319/1,519). The median ages of cases with mild, moderate, and severe dehydration were 25.0 years, 27.0 years, and 29.0 years, respectively. The Kruskal–Wallis test was used to compare these medians and indicated a significant association between increasing age and an increase in the severity of dehydration (p-value < 0.0001).

### Seasonal variation of cholera cases during the 2018–2023 outbreaks in Cameroon

Apart from the Sudano-Sahelian zone which experienced higher case-notification in the dry seasons, all the other sub-climatic zones experienced higher case notification during the rainy seasons. Peaks occurred during the rainy and dry seasons but overall, more cases were notified during the rainy seasons. Figure 3 (Additional File 3) describes the variation of number of cases reported during different seasons in each climatic subzone during the study period.

### Factors associated with mortality during the 2018–2023 cholera outbreaks in Cameroon

On bivariate analysis, there were significantly higher odds of death among cases notified in rural health areas than among those notified in urban health areas (OR 1.68, *95% CI: 1.38–2.03*) and significantly higher odds of death among male cases (OR 1.97, *95% CI: 1.70–2.28*). We found significantly higher odds of death among cases older than 35 years, with the highest odds in persons older than 65 years (OR 4.29, *95% CI: 2.17–6.77*). Severe dehydration, (OR 13.7, *95% CI: 8.28–22.70*) also appeared to be significantly associated with increased cholera mortality. On multivariate regression, age greater than 45 years, severe dehydration, dry season and male sex were predictors of increased cholera mortality (Table 3).

**Table 3:** Bivariate and multivariate analyses of factors associated with cholera mortality in Cameroon, 2018–2023

Factors	Category	Clinical outcome of cases		Bivariate OR (95% CI); p-value	Multivariate aOR (95% CI); *p*-value of aOR
		**Alive**	**Dead**		
**Type of health district**	Urban	11,502	261	1	1
	Rural	4,879	186	1.68 (1.38–2.03); *p* < 0.0001	1.07 (0.84–1.37) *P* = 0.594
**Sex**	Female	7,729	447	1	1
	Male	9,749	306	1.97 (1.70–2.28); *p* < 0.0001	1.61 (1.30—2.04) *p* < 0.0001
**Age group (years)**	0–4	1,647	28	1	1
	5–14	2,227	48	1.27 (0.79–2.02); *p* = 0.3217	0.78 (0.46–1.35); *p* = 0.38
	15–24	3,786	48	0.75 (0.46–1.19); *P* = 0.2193	0.59 (0.35–1.00); *p* = 0.049
	25–34	3,885	74	1.12 (0.72–1.74); *p* = 0.6113	0.75 (0.45–1.23); *p* = 0.25
	35–44	2,545	90	2.08 (1.36–3.19); *p* = 0.0006	1.38 (0.08–2.24); *p* = 0.194
	45–54	1,571	65	2.43 (1.55–3.81); *p* < 0.0001	1.66 (1.01–2.75); *p* = 0.047
	55–64	871	58	3.92 (6.48–6.20); *p* < 0.0001	2.58 (1.52–4.35); *p* < 0.0001
	≥ 65	810	59	4.29 (2.71–6.77); *p* < 0.0001	2.37 (1.38–4.05); *p* = 0.002
**Level of dehydration**	Mild	3,121	16	1	1
	Moderate	7,612	45	1.15 (0.65–2.04); *p* = 0.625	1.17 (0.66–2.09); *p* = 0.58
	Severe	4,482	315	13.7 (8.28–22.70); *p* < 0.0001	12.76 (7.66–1.25); p < 0.0001
**Season**	Rainy	13,169	369	1	1
	Dry	3,310	109	0.85 (0.69–1.10); *p* = 0.144	1.67 (1.28—2.22); *p* < 0.0001

## Discussion

### Summary of main findings

This study described the cholera epidemics that occurred in Cameroon between May 2018 and March 2023. It also investigated factors influencing mortality from cholera during this period. Earlier studies have described previous cholera outbreaks and associated factors of susceptibility and mortality from cholera but few, if any, have described an analyzed the recent outbreaks from 2018 to 2023.

In this study, we found that from 2018 to 2023, about 18,986 cases of cholera were reported in eight out of ten administrative regions in Cameroon. There were four distinguishable cholera outbreaks during this period, with the outbreak from 2021 to 2023 being arguably the largest outbreak ever recorded in Cameroon with about 15,111 cases reported. Three regions, the only regions in Cameroon with a coastline (the Littoral, South and South-West Regions) reported 84% of all the cases. Overall CFR was 2.7% and decreased progressively with each successive epidemic from 2018 to 2023. Age above 45 years, severe dehydration at time of consultation, male sex and cases reported during the dry seasons were significantly associated with increased odds of dying from cholera during this period.

### Interpretation of key findings

From 2018 to 2023, the study period, Cameroon reported a significant number of cholera cases (about 18,986). This trend was similar in its neighboring countries like Nigeria (which reported upward of 100,000 cases between 2021 and 2023), and The Democratic Republic of Congo which reported upward of 52,000 cases in 2023 alone. Contrarily, four out of the six of Cameroon’s neighbors have not reported cases of cholera since 2018. Chad last reported cases in 2017 at its border with South Sudan, Central African Republic last reported in 2016–2017, Equatorial Guinea last reported in 2005 and Gabon lastly reported cases in 2003 [29–32]. These findings highlight the critical importance of strengthening cross-border surveillance of cholera in order to minimize the risk of reintroducing cholera into neighboring countries which are currently cholera-free.

The North and Far North Regions in Cameroon, reputable for being frequently affected by cholera epidemics, accounted for only 11.1% of cases within this period while the South-West, Littoral and South Regions (the coastal regions) accounted for 84% of cases. Comparatively, from 2004–2013 the two northern regions accounted for 47.3% of the cases while the coastal regions (Littoral, South, South-West) accounted for 38.7% of cases [33]. Despite this shift in distribution of cases, the two northern regions and the three coastal regions accounted for the majority of cases put together. Generally, coastal areas are prone to cholera epidemics associated to the survival of *Vibrio cholerae* in marine organisms such as zooplankton [34, 35]. This, compounded by the overcrowding and inaccessibility to potable water in cities like Douala in the Littoral Region and cross-border movements between South-East Nigeria and the South-West Region of Cameroon due to political unrest could explain the high susceptibility of these Regions to cholera [36]. Moise Ngwa et al*.* have linked the frequent cholera epidemics in the northern regions of Cameroon to socio-cultural, economic and climatic factors particularly inaccessibility to clean water due to frequent droughts, mistrust in public health messages (due to multiplicity of ethnic languages, dependency and sentimentality toward animal husbandry), cultural practices of eating food from one plate and hospital avoidance (due to belief that traditional healers cure cholera) [37].

The high number of cases of and the wide geographical spread could be attributed to a multitude of factors with the poor state of water sanitation and hygiene (WaSH) in Cameroon being arguably the most significant. Urban and rural areas alike all face difficulties accessing potable water, large cities are overcrowded with poor drainage systems while rural areas even though less populated, have lesser access to proper sanitation and hygiene infrastructure [25]. The high number of cases recorded especially during the 2021–2023 outbreak could also be attributable to the overwhelming of the health system by the COVID-19 pandemic which led to diversion of already limited resources toward surveillance and response toward COVID-19, negatively impacting cholera surveillance. An additional factor which cannot be overlooked is the number of Regions in Cameroon impacted by humanitarian crises. As previously detailed in the study settings chapter, armed conflicts and terrorist insurgencies within Cameroon and in neighboring countries such as Central African Republic and Nigeria have led to a significant increase in refugee resettlement in Cameroon and also a marked increase in internal displacement. Regions such as The Far-North, North, East, South-West, and towns with high number of resettled internally-displaced persons (towns such as Limbe, Douala, Yaoundé) were heavily affected by cholera during our study period. The ensuing living conditions (overcrowding, poor water access, poor sanitation and hygiene) in these places could have played a role in the exacerbation (spread) of cholera outbreaks once started. Two regions (North-West and Adamawa) also impacted by humanitarian crises did not report any cases probably because there were not any initial cases that could have been quickly spread.

Despite significant improvements in cholera surveillance and early notification of cases, susceptibility is still high because preventive measures and strategies remain inadequate. For example, oral cholera vaccination strategies in Cameroon and several countries in the sub-region are overwhelmingly reactive rather than pre-emptive. Also, the vaccination coverage even among target populations is still quite low. Inadequate WaSH in overcrowded urban areas and in rural areas, low vaccination coverage, delayed access to treatment, insufficient healthcare services, changing climatic conditions and other factors all combine to leave the population extremely susceptible to cholera and can explain the large numbers of cases notified during recent outbreaks in Cameroon and Sub-Saharan Africa at large. Tackling these multitude of factors involves developing and implementing specific policies and strategies and is evidently not only the responsibility of Ministry of Public Health of Cameroon. Ensuring access to safe water, proper waste management, high vaccination coverage, improved access to healthcare and quick notification systems requires a robust interconnected multisectoral approach involving at least the ministries in charge of public health, animal health, environmental health, agriculture, education territorial administration, and the ministry in charge of housing and urban development.

Even though females made up 50.1% of the Cameroonian population between 2018 and 2023 [38], males appeared more susceptible than females to cholera (male: female ratio of 1.27). Our findings are similar those of recent studies in Haiti, Sierra Leone and Nigeria [39–41]. However, there is not enough evidence to attribute the sex disparity among cases to physiological causes. Rather, socio-cultural (gender) roles played by males and females may explain this finding [41]. The higher rate of male deaths from cholera found in our study has also been cited by authors in Nigeria and Zimbabwe [41, 42]. However, there was no satisfactory explanation that could be offered for the higher odds of death among males. This finding needs to be explored further taking into consideration the the socio-cultural realities of affected communities.

The CFR throughout the period were all significantly higher than the less than 1% WHO threshold [43]. Data from other sub-Saharan countries indicate that similarly high CFRs during cholera outbreaks is the norm rather than the exception as an analysis of cholera patterns and trends in Sub-Saharan Africa from 2000 to 2023 indicated an average CFR of 2.3% [44] Between 2022 and 2024 only three out of eighteen countries in the WHO African Region that had cholera outbreaks recorded CFRs less than 1% (Burundi, Mozambique and South Sudan) [43]. High CFRs during outbreaks could be explained by several factors, one of them being delays in seeking for treatment. Cholera has a very short incubation period, ranging from a few hours to a few days, with rapid onset of severe dehydration. Appropriate rehydration and other supportive measures have to be commenced very quickly after onset of symptoms in order to prevent death from cholera but the current health system infrastructure in Cameroon and many Sub-Saharan African countries is inadequate to provide such services. Another contributor to the high CFRs could be low cholera vaccination coverage because vaccination not only reduces susceptibility to the disease but also reduces severity of disease once infected [45].

Case-fatality increased with age, with the age group age above 45 years presenting a significant risk of mortality from cholera, similar to reports from Global Task Force on Cholera Control [46]. Many recent epidemics in sub-Saharan Africa have shown similar patterns [41, 46, 47]. Dalhat and collaborators, in Nigeria suggested that some possible reasons for this increased mortality among the older population could be lack of proper care, dependence on family members for support or presence of other health conditions and undernutrition [47]. These findings may be explained by the reluctance or difficulty in displacement of older people to seek care in treatment centers. This is a hypothesis that has to be further evaluated as it may significantly reorientate the public health response during cholera epidemics. Cholera outbreak response strategies should be adjusted to tackle this susceptible group. Another hypothesis which may require further exploration is the possibility that increased mortality among the older age groups as observed may be related to the higher prevalence of pre-existing non-communicable diseases such as diabetes, heart disease and chronic kidney disease amongst others. It is possible that diseases such as diabetes and heart disease which are already proven causes of a weakened immune system may also increase susceptibility to cholera and increased cholera severity, hence increased mortality in older people, or that conversely, dehydration and acidosis that occur during cholera may lead to clinical decompensation of these pre-existing pathologies. The CFR among children less than 5 years of age during this period was low despite this group being particularly vulnerable to cholera. Children are generally more prone to death from cholera infection due to difficulty in the clinical management (e.g. fluid management) of pediatric cholera cases as well as their higher susceptibility to malnutrition which exacerbates the risk of cholera-related death, especially in children aged less than five years.

Irrespective of zone-specific climatic conditions, more cases were reported in the rainy season than in the dry season. Cholera data analysis in Cameroon from 2004–2012 is supportive of these findings as it reveals that cholera cases surged mostly during the rainy seasons and at the end of the dry seasons [33]. A kenyan study showed that depending on the time of the year, decreased rainfall could lead to more cases but also, increased rainfall leading to floods also led to an increase in cholera cases [48]. On multivariate analysis, surprisingly, the dry season appeared be associated with higher mortality from cholera. The closest to this we could find in literature, was a modelling study in Iran which showed that low precipitation combined with high temperatures (as observed during the dry season) were associated with an uptick in cholera cases and in which the authors found that *Vibrio cholerae* replicates faster under these conditions [49].

### Study limitations

The cholera linelists retrieved from the Public Health Emergency Operations and Coordination Center (PHEOCC) were missing considerable amount of data for several variables. However, the database was large, with the available data still allowing for enough statistical power. Pairwise deletion was preferred in the bivariate analysis in order to minimize the effect of missng data and was also attempted in the multivariate analysis, but led to inaccurate regression models. Binary logistic regression analysis (multivariate analysis) was therefore conducted with a smaller subset (about 80 percent) of the total number of cases as only cases with available complete data.

During this period, rural areas may have been underreporting cases (and consequently, related-deaths) leading to the finding of a significant disparity between the number of cases reported in urban and rural areas as noticed in our study. This reporting bias may not have been limited only to rural areas, but also to deaths occuring outside of health facilities (in the community) in both rural and urban areas and could potentially have led to an underestimation of the total number of cholera cases.

Obtaining and harmonizing data in the line-lists from all involved regions and districts was a significant challenge faced by the authors. The authors compared linelists obtained directly from health districts with those obtained from the PHEOCC in order to identify and rectify incoherences before compiling all the data into one database for analysis. Finally, the data did not also permit evaluation of some important factors of cholera death and severity such as time lapse between onset of symptoms and consultation, and also place of death of cases (community or health facility).

## Conclusion

The findings of this study indicate that from 2018 to 2023 Cameroon recorded a high number of cholera cases throughout a large portion of its national territory. The study also affirms increased susceptibility of males, older age groups, severe dehydration and the dry season as factors associated with increased odds of death from cholera. It suggests that reducing the frequency and severity of outbreaks requires the adoption of multisectoral policies and strategies to improve health interventions such as vaccination, water sanitation and hygiene and reducing delays to healthcare access.

## Supplementary Information

Additional file 1: Fig. 1. Administrative regions and health districts from which cases of cholera were notified between May 2018 and March 2023 (the study period). Administrative regions from which cases were reported are shaded light blue. Districts from which cases were reported are shaded dark brown.

Additional file 2: Fig. 2. Epidemic curves and distribution of cholera cases in health districts from 2018–2023 in Cameroon. The maps in the bottom row indicate the districts affected during the corresponding epidemics indicated in the epidemic curves in the top row. The arrows in the maps indicate the evolution of the spread of cases by region/districts affected. This information was generated from the dates of onset of cases notified in the cholera database.

Additional file 3: Fig. 3. Variation of number of cholera cases by season in different climatic subzones in Cameroon, 2018–2023. This classification of climatic subzones and the periods of dry and rainy seasons were obtained from the scientific publication by Cornelius et al. (21).

## Data Availability

All data generated or analyzed during this study are included in this published article [and its supplementary information files].

## Abbreviations

CFR: Case-fatality rate
PHEOCC: Public Health Emergency Operations and Coordination Center
QGIS: Quantum Geographic Information System
MOH: Ministry of Public Health
WaSH: Water Sanitation and Hygiene
