# Supplementary figures and images for: Trends of cholera epidemics and associated mortality factors in Cameroon: 2018–2023: a cross-sectional study
Source: BMC Public Health. 2025 May 16;25:1816. doi: 10.1186/s12889-025-23007-5 (PMC12082990; doi:10.1186/s12889-025-23007-5)

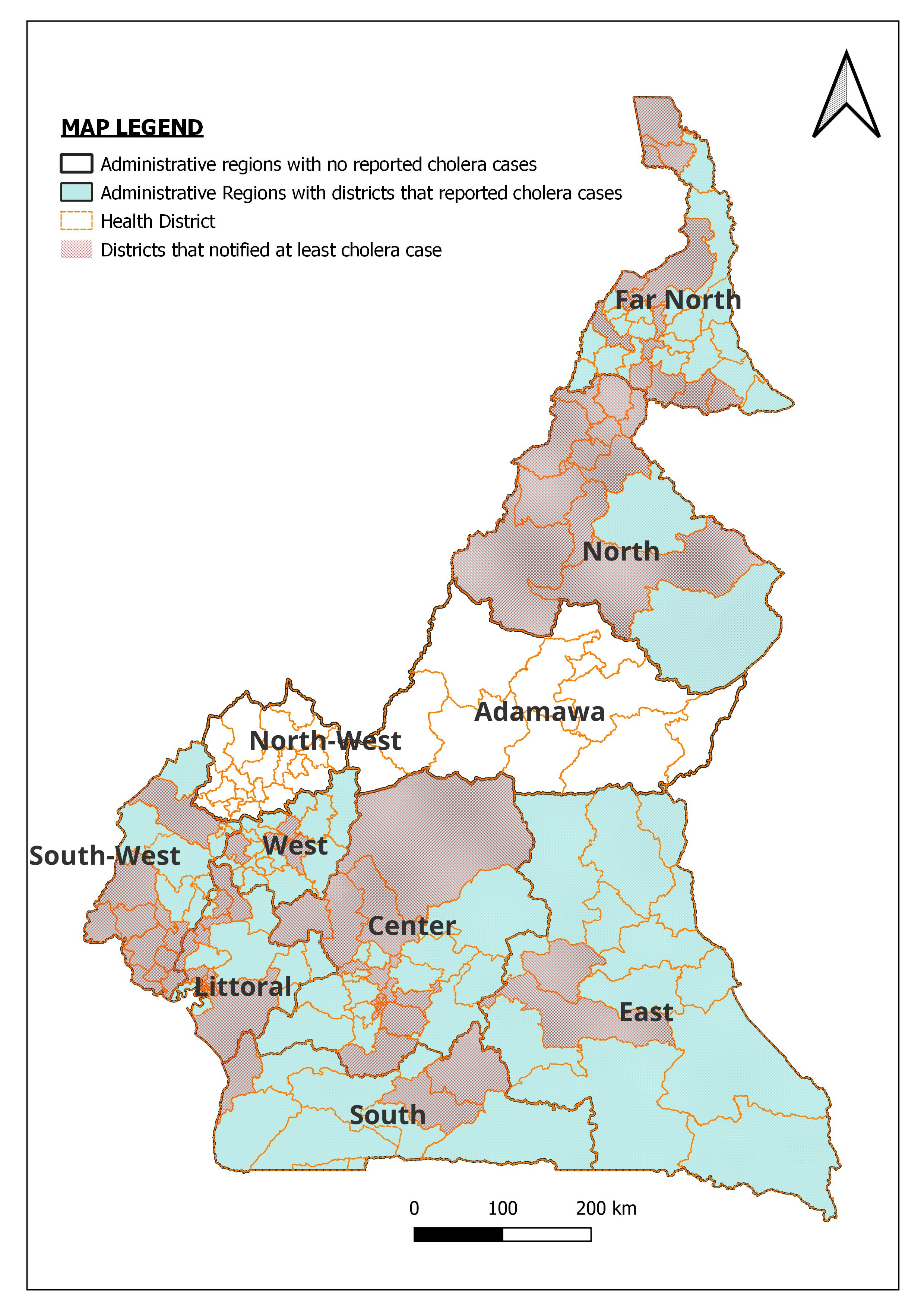

Supplement: Supplementary file 1 — Additional file 1: Fig. 1. Administrative regions and health districts from which cases of cholera were notified between May 2018 and March 2023 (the study period). Administrative regions from which cases were reported are shaded light blue. Districts from which cases were reported are shaded dark brown. [file 12889_2025_23007_MOESM1_ESM.tiff]

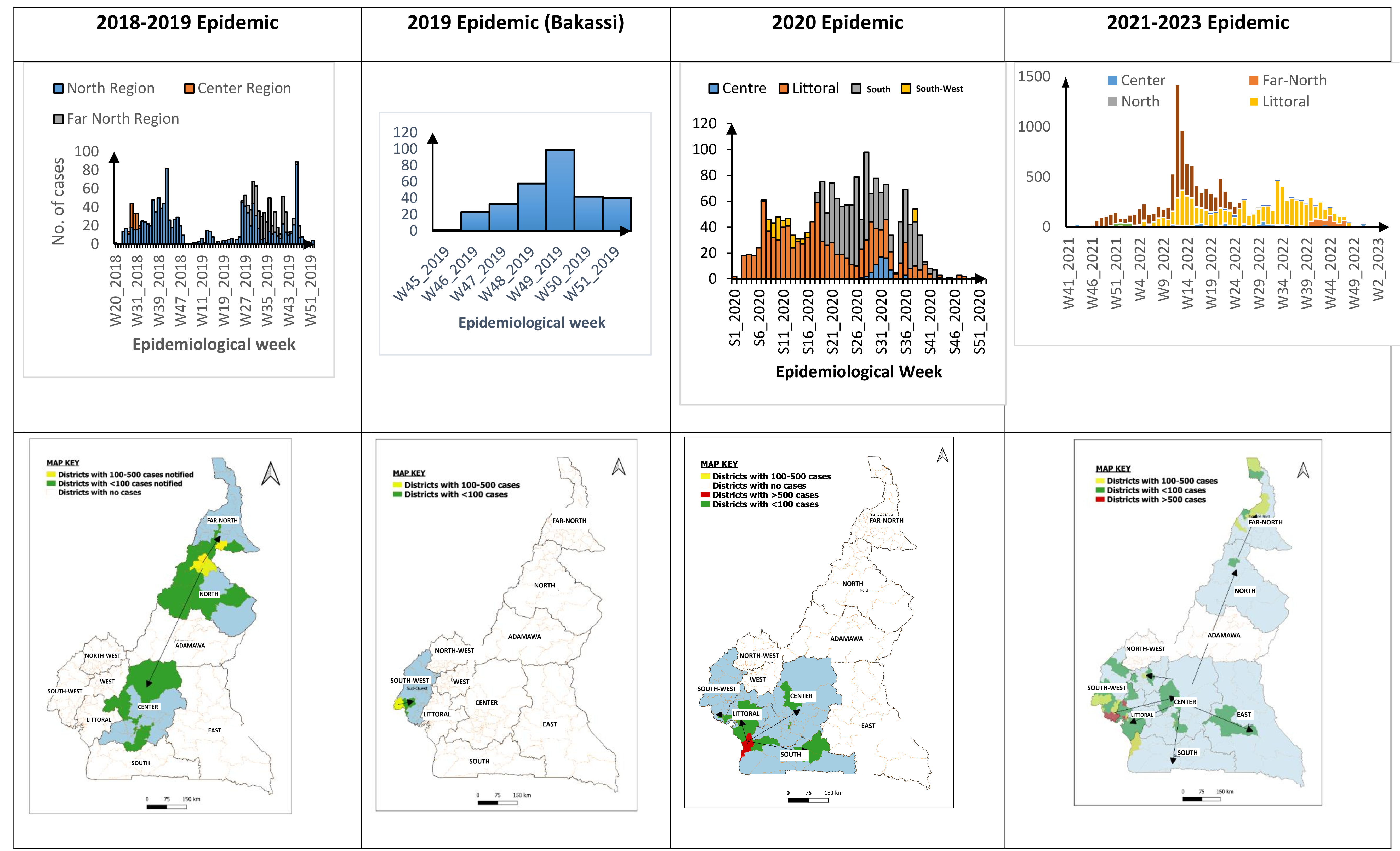

Supplement: Supplementary file 2 — Additional file 2: Fig. 2. Epidemic curves and distribution of cholera cases in health districts from 2018–2023 in Cameroon. The maps in the bottom row indicate the districts affected during the corresponding epidemics indicated in the epidemic curves in the top row. The arrows in the maps indicate the evolution of the spread of cases by region/districts affected. This information was generated from the dates of onset of cases notified in the cholera database. [file 12889_2025_23007_MOESM2_ESM.tiff]

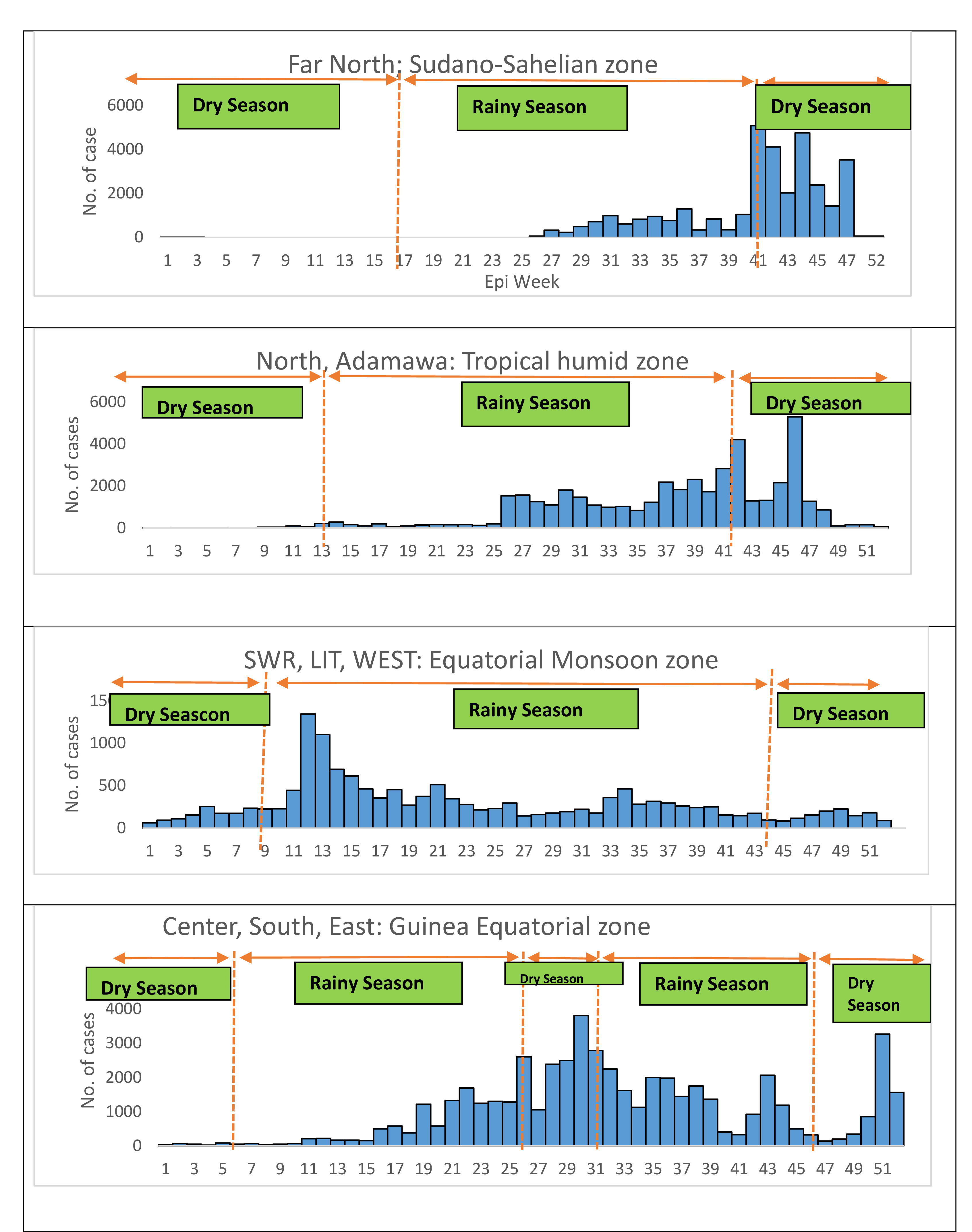

Supplement: Supplementary file 3 — Additional file 3: Fig. 3. Variation of number of cholera cases by season in different climatic subzones in Cameroon, 2018–2023. This classification of climatic subzones and the periods of dry and rainy seasons were obtained from the scientific publication by Cornelius et al. (21). [file 12889_2025_23007_MOESM3_ESM.tiff]
